# Return to Nature: Sacred Plants in the *Azerin* Funerary Rite of *Candomblé Nação* Angola

**DOI:** 10.3390/plants15020260

**Published:** 2026-01-14

**Authors:** Thaís Salatiel de Azevedo, Ygor Jessé Ramos, Nina Claudia Barboza da Silva

**Affiliations:** 1 Postgraduate Program in Plant Biology, State University of Rio de Janeiro-UERJ, Rua São Francisco Xavier, Maracanã, Rio de Janeiro 20550-013, Brazil; thais.salatiel@gmail.com; 2 Laboratory of Farmácia da Terra, School of Pharmacy, Federal University of Bahia-UFBA, Av. Milton Santos, s/n°, Salvador 40170-115, Brazil; 3 Laboratory of Applied Botany, School of Pharmacy, Federal University of Rio de Janeiro-UFRJ, Av. Carlos Chagas Filho, 373, Cidade Universitária, Rio de Janeiro 21941-853, Brazil

**Keywords:** ethnobotany, sacred flora, spiritual protection, *Egun*, Afro-Brazilian religiosity, biocultural heritage

## Abstract

During the transatlantic diaspora, enslaved Africans brought cultural and religious elements to Brazil, such as *Candomblé*, that deeply influenced local society. In *Candomblé* communities of Bantu origin, the death of an initiate requires specific rites, such as the *Azerin*. In this ritual practice, sacred plants are selected according to their associations with deities and their capacity to provide spiritual protection. Each plant used holds particular meanings and is deliberately chosen to create an atmosphere of respect and reverence for the deceased. This study analyzes the symbolism and ritual functions of plants in the *Azerin* funeral rite of *Candomblé Nação* Angola, with emphasis on their role in spiritual protection. Participant observation and review of specialized literature were employed to describe the rite and its vegetal repertoire. Five sacred plant species were identified (*Elaeis guineensis* Jacq, *Raphia vinifera* P.Beauv., *Dracaena fragrans* (L.) Ker Gawl., *Lagenaria siceraria* (Molina) Standl. and, *Morus nigra* L.), whose functions range from protection against *Eguns* to the material representation of the initiate’s body. The results indicate that, in the *Azerin*, plants do not merely express protection but operate as mediating elements in communication with ancestors, structuring the greeting and continuity of ancestor worship in *Candomblé Nação* Angola. The study thus expands the understanding of ritual plants in Afro-Brazilian religiosity and the biocultural heritage of these communities.

## 1. Introduction

The forced displacement of African populations to Brazil during the transatlantic slave trade entailed not only the violent exploitation of labor but also the transplantation of complex cultural, religious, and botanical knowledge systems. Enslaved peoples from regions corresponding to present-day Angola, Congo, Cabinda, Benguela, and Mozambique, commonly grouped under the broad ethnonym “Bantu”, played a central role in the formation of Afro-Brazilian identities and religious landscapes [1,2]. Until at least the mid-seventeenth century, Bantu groups comprised the majority of the enslaved population in different parts of colonial Brazil, decisively shaping local social organization, ritual practices, and cosmological interpretations of life and death [1,3,4].

Among the most enduring legacies of this Bantu presence is *Candomblé*, an Afro-Brazilian initiatory religion structured around the veneration of deities and ancestral forces. In *Candomblé Nação* Angola [free translation: *Candomblé of the* Angola *nation*], these divine beings—the *Nkisis*—are closely associated with elements of the landscape, meteorological phenomena, and specific plant assemblages, and their myths and rites are transmitted predominantly through oral tradition [2,5,6]. The ritual life of the *terreiro* is profoundly embedded in the vegetal world: leaves, roots, barks, and fruits are mobilized in offerings, baths, fumigations, and spatial demarcations, and thereby codify cosmological principles in tangible, sensorial forms [7,8,9,10].

Within this broader ritual repertoire, funeral ceremonies occupy a privileged position as moments in which the relationship between the living and the dead is publicly renegotiated. In *Candomblé* houses of Bantu origin, the death of an initiate requires the celebration of a complex set of rites known as *Azerin*, *Ntambi*, or *Mukundu*, which aim to “untie” the bonds between the deceased, their personal *Nkisi*, and the community [11,12,13]. In these ceremonies, the *ori* shrine—the material locus of the person’s spiritual link with the deity—is ritually dismantled, the *Egun* (ancestral spirit) is guided from *Àiyé* (Earthly Realm) to *Òrun* (Spirit World), and the status of the deceased within the lineage of ancestors is collectively redefined [14,15,16]. The temporal extension of these rites, which may be celebrated in the year of death or many years later, underscores their centrality for the maintenance of hierarchy, memory, and communal cohesion in *Candomblé Nação* Angola [12,17].

Ethnobotany provides an appropriate theoretical and methodological framework to address these phenomena. Classically, ethnobotany has been defined as the study of the interactions and reciprocal relationships between plants and human societies, encompassing all dimensions in which plants are perceived, classified, used, and symbolically elaborated [18,19,20,21]. Rather than focusing solely on utilitarian or pharmacological aspects, contemporary ethnobotanical research emphasizes that plants and people constitute co-constructed systems in which vegetal species act as mediators of identity, memory, and power within specific cultural and ecological contexts. In religious settings, and particularly in Afro-diasporic traditions, ritual plants structure cosmologies and ritual efficacy, shaping not only healing practices but also rites of passage, initiation, and funerary liturgies [9,10,22].

From this perspective, the relationship between *Candomblé Nação* Angola and its sacred plants can be understood as a specific case of ritual ethnobotany, in which botanical species do not merely supply raw material for medicines but serve as active agents in the organization of space, the regulation of spiritual traffic, and the materialization of abstract concepts such as *axé* (spiritual force), *Ìkú* (Death, as a spiritual entity), and ancestrality [See definitions and details at [8,22,23,24]]. In funeral contexts, the selection, arrangement, and manipulation of plant species are key to delimiting sacred boundaries, mediating the passage between worlds, and protecting participants from potentially disruptive spiritual forces. The *Azerin* rite exemplifies this process of drawing on local biodiversity, but it carries precise ritual attributions, emic classifications, and mythical associations that organize the liturgy and shape the relationship between the community and its dead. However, the literature has not described how the ethnobotanical relationships of the ritual are established [7,9,14,25].

Investigations into plant–people interactions in such contexts are therefore fundamental not only for documenting traditional knowledge but also for supporting the sustainable use of biodiversity and the safeguarding of biocultural heritage. Ethnobotanical studies that integrate botanical, symbolic, and social dimensions can help local communities negotiate and strengthen their heritage, inform conservation strategies, and inspire the development of new plant-based foods, phytotherapeutic preparations, and other durable materials of vegetal origin grounded in traditional knowledge [18,19,20,26,27]. In the case of Afro-Brazilian religions, this task acquires particular urgency because *terreiros* are often located in urban peripheries marked by environmental vulnerability and are simultaneously exposed to religious racism, institutional stigmatization, and pressures from urban redevelopment [3,6,16,28]. Under these conditions, the plants, spaces, and ritual practices that sustain *Candomblé Nação* Angola become especially fragile components of Brazil’s biocultural diversity.

Despite the growing ethnobotanical literature on Afro-Brazilian religions, most studies have privileged therapeutic uses of medicinal plants, initiation rites, or more widely known liturgies, particularly in Ketu and Jeje traditions [9,10,22]. The ritual uses of plants in funerary ceremonies of Bantu-Angola matrices remain comparatively underexplored, and systematic data on species, vernacular names, and ritual functions in rites such as *Azerin* are scarce. Furthermore, few works explicitly discuss sacred plants in these contexts as elements of biocultural heritage, situated at the intersection of religious practice, environmental management, and struggles against religious intolerance in contemporary Brazilian cities [4,6,16].

This article addresses these gaps by presenting a detailed ethnobotanical analysis of sacred plants employed in the *Azerin* funeral rite of *Candomblé Nação* Angola. Building on participant observation of a complete *Azerin* ceremony and botanical documentation of the species involved, we (a) describe the ritual sequence with attention to the spatial and temporal positioning of plants; (b) identify and systematize the botanical species and vernacular names used, as well as their main symbolic and ritual functions; and (c) interpret how these plants structure the passage of the *Egun* from *Àiyé* to *Òrun*, with emphasis on their protective, purifying, and commemorative roles. We hypothesize that, in the *Azerin* context, the symbolic use of plants is oriented primarily toward spiritual protection and the management of the presence of *Ìkú*, while simultaneously reaffirming ancestral continuity and the cosmological centrality of nature in *Candomblé Nação* Angola [14,15,22]. By registering this repertoire in a systematic way and situating it within broader debates on biocultural diversity and religious racism, the study contributes to Afro-Brazilian ethnobotany and to the recognition of sacred plants as key components of urban biocultural heritage.

## 2. Results and Discussion

In *Candomblé*, life is celebrated just as much as death. The deceased is honored with food, drinks, songs, and dances in the rituals of *Azerin*, *Mukundu, Sirrum, Axexê*, or *Ntambi*. Regardless of the *Candomblé* nation in which the funerary ritual is performed, all participants wear white, as white clothing in this context symbolizes absolute truth, death, and life [16]. During the ritual, five species of sacred plants were observed, whose uses ranged from protection against *Eguns* to the physical representation of the deceased person (Table 1):

The Afro-Brazilian worldview emphasizes that death does not signify total extinction or annihilation. To die is to undergo a change of state, of plane, and of existence. Thus, the initiate, priest, or priestess who successfully fulfills the entirety of their destiny and has had the proper rituals celebrated according to tradition, transforms into an ancestor. They may even be invoked as an *Egun*, becoming part of the *axé* of the *terreiro*’s ancestral lineage. To affirm this recognition, the *Azerin* is performed in the *Candomblé Nação* Angola.

The *Azerin* takes place within seven days after the burial of the deceased *candomblecista* [Free translation: *Candomblé devotee*], marking a period of mourning and reflection. At this time, the footsteps of the *Egun* are swept away, symbolizing the interruption of their return to the world of the living. The ritual unfolds around the central pole, which connects the *Ilê* (sacred house) to the cosmos, highlighting the importance of hierarchy in these rites.

Immediately after the death of the *candomblecista*, when the body is handled by the priests, a symbolic mark implanted at the top of the skull during initiation—representing the deity to which the person was dedicated—is removed. The hair in that area is shaved, and the head is washed with a sacred herbal preparation known as *Amassi*.

The leaves used in *Amassi* vary depending on the deity to whom the person was initiated. The elements of this preparation are rarely known outside the priesthood that conducts the ritual. Symbolically, this herbal mixture reverses the first initiation rite, when the head of the newly initiated is likewise washed by the priest or priestess. *Amassi* is the first element used in the offering to honor the deceased during the *Azerin*.

**Figure 1:**
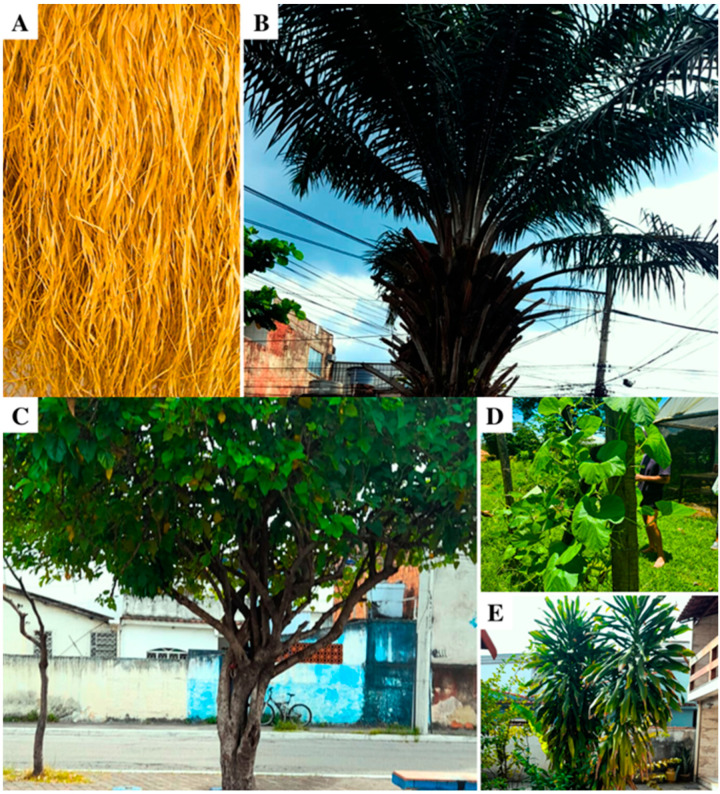
Sacred plants used in the *Azerin*. (**A**) Palha-da-costa (*Raphia vinifera* P.Beauv.); (**B**) Dendezeiro (*Elaeis guineensis* Jacq); (**C**) Amoreira (*Morus nigra* L.); (**D**) Cabaça (*Lagenaria siceraria* (Molina) Standl.); (**E**) Peregum (*Dracaena fragrans* (L.) Ker Gawl.). Image created by the authors.

To begin the ritual, sacred songs are chanted and incense is burned. *Palha-da-costa* (*Raphia vinifera* P.Beauv.) (Figure 1A) is present throughout the ritual. The *Ogãs* wear strips of *Palha-da-costa* on their left wrists as a form of protection against potential approach by *Eguns*. Women participating in the ritual tie *Palha-da-costa* around their heads with the same purpose of warding off the presence of *Eguns.*

A table is prepared with various symbolic elements. Among these elements is the shredded leaf of the oil palm (*Elaeis guineensis* Jacq) (Figure 1B), whose ritual name is *Mariwo*. *Mariwo* is placed on the table for *Egun* with the intention of repelling spirits that may approach with negative intentions, serving as a form of protection for participants in the ritual.

Chants in honor of the ancestors are sung in three *Candomblé* nations, starting with the nation to which the deceased was initiated, followed by songs from the Ketu and Jeje *Candomblé* traditions. After this moment, songs for the mulberry tree and the *Egungun* cult of Itaparica Island (Bahia) are performed as a tribute to the knowledge held by these traditions.

During this ceremony, the *atabaques* (ritual drums) remain silent, a gesture of respect and mourning. Because of this, the traditional *atabaque* is replaced by a large gourd (*Lagenaria siceraria* (Molina) Standl.) (Figure 1D), turned upside down in an earthenware bowl filled with water, cachaça, honey, palm oil, and dendê oil. It is played with sticks made from the branch of the mulberry tree (*Morus nigra* L.) (Figure 1C), known as *Aguidavi*. The gourd is accompanied by a ceramic pot, from which a muffled sound is produced using straw fans beaten on its opening.

Offerings to *Egun* are arranged to symbolize the obligations of the deceased and reflect the traditions of the *Candomblé* nations: Ketu, Jeje, and Angola. On this ceremonial table, various bowls are placed surrounded by bottles of cachaça, with small flags in shades of red, white, yellow, and black. At the front, a candle burns beside six bowls of *Padê* (manioc flour with dendê) and an empty gourd. All these items are covered with a white cloth, adorned with *mariwôs*, giving the space an aura of sacredness and reverence.

The gourd is also used to represent the deceased honored during the *Azerin*. An empty gourd is placed, into which coins are deposited by attendees as each person dances for the *Egun* [25]. This dance is a personal tribute from those present to the deceased. Despite the lively and engaging rhythm of the *Azerin*’s chants and dances, the tone of the celebration is sorrowful, as the songs recall moments that will not return [17].

The *Azerin* may last up to twenty-one days, which not only intensifies the mourning experience but also reaffirms the need for collective introspection and spiritual renewal [17]. At the end of the ritual, an energetic cleansing (spiritual cleansing) of the space and the participants is performed using Peregum (*Dracaena fragrans* (L.) Ker Gawl.) (Figure 1E). This cleansing is conducted by the *Nkisi* Zumbarandá, Kavungo, or Matamba, incorporated into one of the initiated individuals present. When no such initiate is available, an *Ogã* carries out the ritual.

All the materials used during the ritual are disposed of at a location previously determined through the cowrie-shell divination. The *orixá* shrine, a physical object existing in the *Àiyé*, also represents a spiritual link between the worlds of *Òrun* and *Àiyé*, emphasizing the immortality of ancestral memory and the continuity of traditions. Therefore, the fate of this important element is also defined through the divinatory game and is sometimes likewise ritually discarded [25].

Thus, in the *Azerin*, sacred plants symbolize protection and a means of approaching the ancestors, serving as tools for honoring them [25]. For this reason, the species mentioned are considered indispensable for the perpetuation and continuity of ancestor worship in the *Candomblé Nação* Angola.

An ethnobotanical study on the traditional use of these plants could contribute to valuing ancestral knowledge, offering a foundation for the conservation of cultural practices and the strengthening of community identity. Furthermore, the results could inform educational and environmental preservation initiatives, promoting the importance of plants within the ritual context. However, there is a scarcity of information regarding the use of these plants in funeral rites, revealing a gap that must be addressed to ensure the continuity and recognition of this cultural heritage.

Based on the content analysis and the dialog with the literature, three main thematic axes were identified and are discussed in the following subsections: “*Ìsinku: life, death,* life—Funerary cosmology and the life–death–life cycle”, “*Hierarchy and ancestrality*–Ritual authority and community order”, and “*Jinsaba: return to nature*–Sacred plants as mediators between humans and nature”.

### 2.1. Ìsinku: Life, Death, Life—Funerary Cosmology and the Life–Death–Life Cycle

Reflections on the existence beyond life are a recurring theme in many religious traditions, including the Afro-Brazilian worldview. Questions about what happens to the being after this current life are addressed through concepts such as birth (*ìbí*), life (*ìyè*), and death (*Ìkú*), followed by the afterlife (*ìyè lè̟hìn kú*) and divine judgment (*ìdájó̟ ti Ọló̟run*), ultimately leading to the possibility of return in another life (*Atúnwa*) [29]. 

In the context of *Candomblé*, reaching the afterlife requires going through *Ìsinku*, a term that refers to the funeral rites. The manner in which these rites are conducted is shaped by factors such as the deceased’s age, social status, and religious ties. Each ceremony is adapted to the unique life of the individual, giving distinctive meaning to both death and the celebration of life [13,29].

The funeral rite in *Candomblé Nação* Angola, known as *Azerin*, holds a prominent place in the universe of ritual ceremonies, standing out for its uniqueness and spiritual depth. The *Azerin* is reserved for those who have been initiated, emphasizing the importance of initiation and a deep understanding of practices involving ancestor worship. The primary purpose of the *Azerin* is to release the *Egun*, or ancestral spirit, from the inert matter once occupied in earthly life. This moment is crucial for the *filhos e filhas de santo* (initiates), as the performance of this rite reflects an understanding of the transition between the material and spiritual worlds [11,12,29].

According to Beniste [29], “*Egun has no flag*,” meaning that chants are sung not only in the Nation to which the deceased was initiated but also in *Candomblé* Nação Ketu and Jeje as a way of honoring the enslaved ancestors of the African diaspora. As Santos [15] notes, these ancestral celebrations are traditionally led by men, who hold the title of *Ojé*, a designation that implies rigorous preparation and knowledge of traditions. Guiding the *Egun* (soul of the deceased) on their new journey is a profound responsibility, marked by reverence and care [7].

Funeral celebrations in the context of *Candomblé* can be interpreted as “conceptions of death, secrecy, and tradition that reveal a particular way of seeing and understanding the world—in other words, an Afro-Brazilian worldview” [15]. This understanding of death transcends the notion of a mere end, assuming a dimension where eternity is woven into finitude.

In the Afro-Brazilian worldview, *Ìkú* is the personification of death, seen as an agent designated by *Olódùmarè*, the Creator, to take those whose time on Earth has come to an end. Death, referred to as *Ikú*, is an inevitable and unpredictable force, generally acting upon the elderly. The premature death of young people is perceived as a tragedy, highlighting the fragility of life [13,20].

Yoruba cosmogonic myths recount the story of *Oduleke*, whose death illustrates the depth of funeral rites in Afro-Brazilian religions. *Oya*, Oduleke’s adoptive mother, paid tribute by gathering his belongings and, for seven days, dancing and singing in his memory. This ritual culminated in a procession that placed a bundle containing Oduleke’s belongings at the foot of a sacred tree—an act called *Àjèjè*, which later became associated with the rite of *Àsèsè*. Originally restricted to hunters, this practice expanded to encompass the entire community, reflecting the universal need to honor the ancestors [29,30].

One of the *Azerin* chants dedicated to *Oxóssi* in *Candomblé* Nação Ketu exemplifies this deep connection to ancestry and the passage between worlds:

“*Bibi bibi lo bi wá*,

*Ode àrólè lo bi wá*”.

The birth that brought us into the world

*Odé Àrólè* brought us into the world.

Thus, *Azerin* is not merely a farewell ceremony; it is a ritual of connection between *Òrun* and *Àiyé*, a celebration that reaffirms the continuity of life and spirituality [24]. By releasing the *Egun*, the community not only pays tribute to what once was but also reaffirms its belief in the eternity of the soul, perpetuating the Afro-Brazilian tradition that embraces death as an integral part of the life cycle.

### 2.2. Hierarchy and Ancestrality—Ritual Authority and Community Order

In the context of *Candomblé Nação* Angola, it is common to hold festivities in honor of the ancestors. These celebrations aim, symbolically, to reinforce the ties with those who paved the way for the community and who, through their memory, continue to uphold the collective identity. Ancestrality is thus exalted, with the awareness that the spirits of the deceased, especially those who left meaningful legacies, continue to influence and guide the living [31].

In the *Azerin* ritual, the presence of deities such as Zumbarandá, Kavungo, and Matamba is indispensable. Zumbarandá is the personification of primordial creation, emerging before everything—before the Age of Metals. She arises from the calm, muddy waters of a lagoon, bringing with her the essence that enables the shaping of humanity, establishing herself as the Matriarch who represents the essence of being. Beside her, Kavungo stands out as the king of all spirits, ruling with authority over the ancestors who accompany him. Under the shadow of the raffia, Kavungo conceals the mystery of death and rebirth, revealing the genesis of existence and transcendence [32].

Although Zumbarandá and Kavungo are deeply connected to the *Azerin*, it is Matamba who governs this rite. She is the heiress of the red feminine principle, the generative female force that underpins the existence of the *Egun*. Lady of the *òpákòko*—a ritual trunk embedded in the earth, consecrated for the invocation of ancestors—Matamba is thus queen and matriarch of the *Eguns*. She is revered in her sovereignty, ruling the world of the dead and dispatching the spirits with a basket in sacred rituals [31]. This prestige is corroborated by Santos [15], in his work *Os Nagô e a Morte* (*The Nagô and Death*), where he exalts her as Queen and Founder of the Secret Society of *Egúngún*, highlighting her significance in rites of passage.

This relationship between the deities and the dead is not limited to a simple act of transition but also encompasses the maintenance of morality and social balance. Matamba, like other *Nkisi*, acts as an intermediary, connecting the two worlds and ensuring that the ritual order is respected—a responsibility that extends to the members of the community during the ceremony [33].

In this rite, the *Eguns*—or ancestral spirits—occupy a prominent position, especially those who held significant social or religious roles, such as priests. The remembrance and respect for these spiritual beings are expressed during ceremonies not only to honor them but also to ensure they are properly placed in the *House of Egun*, a sacred space located outside the *terreiro* [29].

At the heart of the religious traditions surrounding the *Egungun* Cult on Itaparica Island, Bahia, as explained by Santos [15], lies the uniqueness of a cult strictly led by men, in which only male spirits, from both Brazilian and African heritages, are venerated. In contrast, within the *Azerin* cult, reverence for *Eguns* transcends such gender restrictions, encompassing not only male ancestors but also those of female lineage, whose contributions were essential to the continuity and richness of this cultural manifestation.

It is important to note that the complexity of funeral rites varies depending on the level of initiation of the deceased. The deeper the connection with the tradition, the more intricate the rituals to be performed. This characteristic demonstrates the direct relationship between the time of initiation and the depth of the bonds formed in *Àiyé*, the world of the living [29].

The hierarchy of *Candomblé* is reflected not only in the way rituals are conducted but also in the social organization they imply. The death of an authority figure not only alters the dynamics of the *terreiro* but also initiates a period of expectation and reassessment of roles within the community. Consultation with *Ifá* becomes essential to determine who will next occupy the position, thus emphasizing the importance of lineage and the recognition of individual contributions throughout the group’s history [13].

*Azerin* does not deal solely with death but also with life and the way the living relate to their ancestors. The ritual becomes a space for reaffirming collective identity, where the bonds that unite past, present, and future are reestablished. The presence of the *Egun*, symbolizing the memory of those who have passed, is celebrated and revered, reaffirming that life is a continuous cycle in which ancestry plays a crucial role in the construction of being and belonging [12].

Thus, the funeral rite of *Candomblé*, through the *Azerin*, represents a network of meanings that encompasses hierarchy and ancestrality—fundamental elements in maintaining culture, tradition, and the collective identity of a community that values the continuity of its history and reverence for its ancestors [34].

### 2.3. Jinsaba: Return to Nature—Sacred Plants as Mediators Between Humans and Nature

Afro-Brazilian religions, with their rich oral tradition and reverence for ancestry, incorporate into the *Azerin* ritual the liturgy of nature, using plants which, according to Durkheim [23], are endowed with sacred value. This value grants plants a supernatural dimension, allowing them to become instruments of healing and purification. The sacredness of plants is manifested in the belief that their biological characteristics can be activated by healers and priests, creating an intimate connection between rituals and the healing power of the plant kingdom.

The funerary rite known as *Azerin*, like other rituals in *Candomblé*, also demonstrates the connection between human beings and nature, expressing the search for a spiritual link that transcends material life. This ritual highlights the veneration of the ancestors and emphasizes the central role of plants as mediators between the physical and spiritual worlds [9].

By honoring the *Eguns*, or ancestral spirits, *Azerin* reaffirms the belief in the continuity of life after death, promoting the liberation of the spirit from the matter that binds it. Thus, the symbolism of plants in *Candomblé* is, as Camargo [22] aptly observes, constructed from myth and legitimized through ritual, imbuing them with powers that emanate from supernatural forces.

In line with Camargo’s [22] observation, according to Beniste [14], in *Candomblé*, the gourd (*L. siceraria*) has various uses. It can serve as a container for ritual baths, be part of the “delivery of the bowl” ceremony (when the tools are handed to newly ordained priests authorized to open their own houses), adorn deities, and appear in funerary rituals. The shape of the gourd is associated with the perfect representation of the world, when *Òrun* and *Àiyé* were still connected, since in the beginning there was no separation between heaven and earth.

During *Azerin*, the leaves and herbs used are not merely decorative elements but essential components that enhance the efficacy of the ritual. They represent the connection between the world of the living and the dead, facilitating the transition from *Àiyé* to *Òrun*. This duality underscores the mediating function of plants, which are considered carriers of *axé*, the vital energy that permeates all things [14].

Verger [8] emphasizes the importance of leaves, noting that, at the time of initiation, contact with plants is fundamental for the absorption of *axé*. This concept extends to *Azerin*, the moment of departure, where the presence of plants becomes indispensable to the performance of the rite. Before the ceremony, participants often undergo herbal baths, seeking purification and the incorporation of the energies that these plants offer. This practice demonstrates the belief that nature, when invoked, can facilitate the spirit’s transition, promoting a deeper connection with the departed loved ones.

Therefore, *Azerin* is not merely a farewell ritual but a celebration of the continuity of life, in which plants play an essential role. They act as bridges linking the material to the spiritual, allowing the *Eguns* to free themselves from earthly bonds and return to their origin [12]. In this way, the worship of nature, so intrinsic to Afro-Brazilian traditions, finds in each leaf and each root the possibility of healing and renewal, perpetuating the ancestral wisdom that brings us back to nature.

### 2.4. Ethnobioconservation and Heritage Policies

Viewed through an ethnobioconservation lens, the five species mobilized in *Azerin* configure a compact yet highly structured biocultural assemblage that condenses diasporic histories, ritual functions, and ecological relations. *E. guineensis* and *R. vinifera* are palms of African origin, intimately associated with the Atlantic slave trade and Afro-diasporic foodways and ritual practices, while *D. fragrans* is likewise an African taxon that has become a ubiquitous ornamental in Brazilian home gardens and *terreiros*. *L. siceraria*, an Old World domesticate with deep funerary symbolism, and *M. nigra*, of Eurasian origin, illustrate how non-African exotics are re-signified within Afro-Brazilian cosmologies, acquiring roles as ritual instruments and ancestral mediators. Together, these species exemplify what studies of *Candomblé* ethnobotany have repeatedly shown: sacred plant repertoires are not static “African relics”, but dynamic Afro-Atlantic constructions in which African, American and cosmopolitan taxa are continuously combined, substituted and resignified in response to historical and ecological constraints [35,36]. In this sense, the plant set documented for *Azerin* is both botanically specific and representative of broader biogeographical processes that underpin Afro-Brazilian religions.

From an ecological perspective, none of these species is globally threatened, but their local availability is strongly mediated by the ways in which *terreiros* manage green areas, home gardens and access to urban vegetation. In many Brazilian cities, sacred yards, “mato” fragments and temple gardens function as micro-reserves where ritual plants are cultivated, protected and exchanged, often with floristic compositions comparable to urban forest remnants [37,38,39]. Urban expansion, gentrification and conflicts over land use—including cases where listed *terreiros* face pressure from real-estate development—tend to reduce the extension and quality of these green enclaves, with direct consequences for the continuity of ritual plant repertoires [40,41]. At the same time, restrictions on accessing surrounding forest fragments or conservation areas, sometimes underpinned by environmental and religious racism, limit the possibility of collecting particular taxa or plant parts needed in rituals [42,43,44,45]. In this scenario, the *Azerin* plant set illustrates a situation of “biocultural vulnerability”: if specific palms, gourds or woody taxa disappear from local landscapes or become legally or socially inaccessible, one of the material pillars of the funeral liturgy is weakened, with ripple effects on knowledge transmission and on the integrity of ancestral worship. This aligns with recent discussions in ethnobioconservation and evolutionary ethnobiology, which emphasize the need to consider intertwined biological and cultural dimensions—and their feedbacks under urbanization and global change—as explicit targets for conservation [46,47,48].

The *Azerin* case study also makes visible the strong interface between ritual and medicinal domains, a pattern widely documented for Afro-Brazilian religions. Surveys in *Candomblé* and Umbanda *terreiros* show that the majority of ritual plants are simultaneously recognized for therapeutic indications and are used in baths, teas, compresses, inhalations and defumations, particularly for minor ailments, spiritual afflictions and “nervous” conditions [35,36,49,50,51]. Within this broader pattern, several of the species highlighted here circulate between medicinal and funerary contexts: *E. guineensis* provides oils and leaves associated with healing, protection and nourishment; *M. nigra* is locally known for nutritional and medicinal properties of its fruits and leaves; *D. fragrans* is central in baths and cleansing rites aimed at removing “negative energies” and restoring balance; and gourds of *L. siceraria* double as containers for herbal preparations as well as symbolic bodies in funerary liturgy. The overlap of medicinal and ritual functions reinforces the argument that these are “key species” for maintaining physical, spiritual and communal health; their loss would impact not only a single rite but a wider network of care practices anchored in plants.

In terms of the anthropology of death and Afro-Brazilian ethnobotany, the detailed description of *Azerin* contributes in at least three directions. First, it shifts attention from the more frequently documented *Axexê* and *Egungun* cults to a Bantu-origin funerary rite in the Angola Nation, showing how a relatively small number of plants can be articulated in a highly specialized choreography of protection, representation of the corpse and cleansing of the ritual space. Second, it reveals a specific configuration in which an Angola rite incorporates chants and references from Ketu, Jeje and *Egungun* traditions, while at the same time maintaining its own botanical and liturgical particularities, thereby evidencing intracultural diversity in the management of sacred flora and conceptions of *Ìsinku* (“life–death–life”). Third, by comparing the gender configuration of ancestors and ritual leadership with classic descriptions of *Egungun* cults in Bahia, in which only male spirits are publicly celebrated, the *Azerin* material nuances the idea of a homogeneous Afro-Brazilian funerary pattern and stresses the plurality of ways in which plants, bodies and spirits are orchestrated in rites of passage [9,12,29]. In sum, a plant-centered reading of *Azerin* complements and expands previous analyses that have privileged drums, masks and shrines, positioning sacred flora as analytical protagonists in the study of death and ancestry.

Framing these findings within the field of heritage and public policy reinforces that *terreiros* are simultaneously temples, seed banks, urban forests and non-formal educational spaces. Studies in Salvador, São Paulo and other Brazilian cities have pointed to *Candomblé* houses as important reservoirs of Atlantic Forest biodiversity and as strategic nodes for urban environmental education, agroecology and the transmission of ecological knowledge [37,38,39,48,51,52,53,54,55,56]. Recognizing, in scientific terms, the specificity of ritual plant assemblages such as those of *Azerin* strengthens demands for policies that protect *terreiros* as biocultural heritage—including legal safeguards for their green areas, support for in situ conservation of ritual species and the explicit inclusion of sacred plants in inventories of urban biodiversity. Conversely, episodes of religious racism, criminalization and symbolic delegitimization of Afro-Brazilian religions—often accompanied by vandalism, territorial pressure and restrictions on traditional plant management—also constitute threats to this ethnobotanical heritage and should be understood as forms of biocultural erosion and environmental injustice [40,42,44,45]. From an ethnobioconservation perspective, safeguarding the plants of *Azerin* implies not only conserving taxa but also defending the religious territories, ritual freedoms and knowledge systems that give them meaning.

In practical terms, the heritagization and long-term sustainability of *Azerin*-related plant knowledge require community-led safeguarding measures that protect both biological resources and the social institutions through which meanings are reproduced [26,27,48]. This includes prioritizing in situ cultivation of key ritual species in *terreiro* green areas and in home gardens [35,36,37,38,39,49]; strengthening culturally regulated exchange networks for plant material [35,36,49]; and supporting non-formal, intergenerational transmission through apprenticeship during ritual preparation and community-based educational activities conducted under local rules of access and secrecy [14,15,35,36,37,38,39]. At the interface with public policy, safeguarding should also translate into concrete protections for *terreiro* territories and their gardens in urban planning and heritage agendas [17,41,42,43], alongside culturally appropriate documentation protocols that prevent extractive use of local knowledge and ensure that any dissemination remains authorized by the community [21,22,23,24,25,26,27,48]. These applied steps align the recognition of *Azerin* as biocultural heritage with feasible mechanisms for continuity, protection, and ethical transmission in rapidly changing urban landscapes [26,27,28,29,30,31,32,33,34,35,36,37,38,39,40,41,42,43,44,45,46,47,48].

### 2.5. Ethnobotanical Cross-Cultural Aspects in Azerin and Study Limitations

Across diverse funerary systems, plants and plant-derived artifacts recurrently operate as materially visible mediators of transition, boundary demarcation, and collective memory, functioning as fuel/structural elements in cremations, offerings in burial contexts, and protective devices that regulate contact between the living and the dead. In *Azerin*, the observed repertoire (*E. guineensis*, *R. vinifera*, *D. fragrans*, *L. siceraria*, and *M. nigra*) falls within this broader comparative framework: leaves, fibers, and vegetal bodies are not merely decorative but are activated as technologies of passage and containment, composing a ritual grammar in which protection, purification, and the materialization of the deceased become inseparable [57,58].

At the Brazilian scale, comparison with ethnobotanical inventories of Afro-Brazilian religious houses indicates both continuity and specificity. Studies in Bahia, Paraíba, and Southern Brazil repeatedly document the centrality of “working with leaves” and the convergence between ritual and protective repertoires, while also evidencing strong intracultural variation in species choice and liturgical choreography. Within this panorama, *Azerin* aligns with the recurrent pattern whereby plants are mobilized to cleanse, protect, and restore ritual balance, yet it is distinguished by the explicitly funerary orientation of its vegetal set and by the prominence of African-origin palms as a formal barrier against *Eguns*; additionally, the combined use of *L. siceraria* and *M. nigra* as material/symbolic operators of the deceased’s presence emerges as novel relative to national datasets in which this pairing is absent or only marginally detailed [49,50,57].

Beyond Afro-Brazilian matrices, part of *Azerin*’s vegetal semantics becomes legible through comparison with Christian practices in Brazil, especially popular Catholicism, in which blessed palms and branches (“ramos bentos”) are widely employed as domestic protectors and prophylactic signs against misfortune, capable of marking thresholds (home, altar, cemetery) and extending ritual efficacy beyond the liturgical moment [53,54]. In *Azerin*, the deployment of palm materials as protective devices and spatial delimiters during the funerary rite resonates with this shared material logic, without presupposing cosmological equivalence, while pointing to convergences that may facilitate pragmatic co-presence in contexts of mixed religiosity.

An additional Afro-Atlantic comparison with Afro-Caribbean traditions (Santería/Ifá in diasporic settings) contextualizes key material forms present in *Azerin* by emphasizing the adaptive circulation of plants and ritual objects across transnational networks and the sustained ritual importance of gourds as containers, instruments, and emblems of spiritual presence, alongside the prominence of leaves and vegetal preparations as technologies of cleansing and protection. Within this broader Yoruba-Atlantic horizon, the use of *L. siceraria* in *Azerin* becomes intelligible as part of an “object–plant” continuum, in which the efficacy of vegetal matter depends on ritual handling, lineage authority, and controlled circulation, rather than on botanical identity alone [49,56].

Taken together, these comparisons support the interpretation that *Azerin*’s botanical assemblage should be understood as a historically stratified Afro-Atlantic construction shaped by diasporic continuities, selective maintenance of African-origin taxa, and pragmatic substitution under local ecological constraints. Similarities with Brazilian and Afro-Caribbean repertoires point to shared logics of protection and cleansing anchored in “leaf-based” ritual technologies, whereas differences—particularly the specific funerary choreography and the concentrated use of African palms as anti-*Egun* barriers—evidence the autonomy of Bantu-Angolan liturgical architectures in Brazil, reinforcing a reading of *Azerin* plants as biocultural operators whose meanings emerge from the interplay among cosmology, lineage transmission, and local availability, rather than as static survivals [49,57].

In funerary and ancestor-oriented liturgies, vegetal repertoires tend to function as “technologies of liminality,” organizing spatial boundaries, purification sequences, and controlled communication with the dead; in this broader context, the *Azerin* assemblage is consistent with an Afro-Atlantic pattern in which leaves, fronds, and plant-derived objects structure passage between domains, yet according to an internal logic centered on the Egum. In Brazil, the most extensively documented intercultural convergence concerns palms and “working with leaves” as protective infrastructure, notably *E. guineensis* (fronds described as apotropaic barriers placed at thresholds) and, in *Azerin*, a more explicitly funerary and sequential use of African palms (including *R. vinifera*) as protective screens against *Eguns*, rather than generalized prophylaxis [49].

Despite the consolidated recognition that plants occupy a structural position in Afro-Brazilian religions, the literature tends to privilege broad liturgical-therapeutic repertoires and general discussions of “sacred leaves,” to the detriment of fine-grained descriptions of the vegetal grammar of specific funerary rites. Classical discussions already emphasized the dynamic maintenance of ethnofloras through retention/introduction of Old World taxa and substitution by locally available species when necessary (“continuity under constraint”); within this framework, the *Azerin* record is innovative in documenting a restricted set of five botanical elements mobilized in a single, clearly delimited funerary rite and in mapping specific functions (delimitation/protection, purification, symbolic substitution of percussion, and performative expulsion of death) [36,49,51,52,55,59].

Macro-regional inventories (e.g., Santa Catarina) reinforce extensive, polyfunctional repertoires and the relational nature of vegetal meaning, whereas evidence from Foz do Iguaçu reiterates contextual liturgical multivocality; species by species, Morus nigra is reported as associated with ancestry in ritual baths, yet in *Azerin* it is operationalized as aguidavi, activated in a sonic-gestural act linked to the expulsion of death, indicating preservation of a shared symbolic nucleus alongside a reorganization of ritual mechanics. Studies in Southeastern Brazil (Minas Gerais) emphasize the centrality of plants and oral transmission; in synthesis, convergences and divergences can be understood through the interaction among the funerary liturgical domain (symbolic densification and functional restriction), diasporic dynamics of availability/substitution, and variability in lineage-specific internal grammars, while reviews indicate persistent gaps in ritual-specific documentation, reinforcing the contribution of empirically grounded, rite-centered records to more precise inter-regional comparisons [36,49,51,52,55,59].

At the same time, some methodological limitations of this study must be acknowledged. The analysis is based on a single *Azerin* rite, observed in a single Angola Nation *terreiro*, and derives exclusively from participant observation and informal emic explanations, without a specific program of formal interviews on funeral practices. The funerary and highly regulated nature of the ritual imposed strict constraints on photographic documentation, on manipulation of plant material and on the degree of detail that could be recorded in field notes, which may have led to under-representation of minor uses or species. The qualitative and interpretative nature of the approach limits statistical generalization; however, the analytical categories and plant–ritual associations described here can be used as hypotheses to be tested in broader comparative studies. Future research could explore cross-national and cross-urban comparisons of funerary rites in different *Candomblé* nations, trace the biographical trajectories of each species from Africa and other regions to Brazilian *terreiros*, quantify the frequency and redundancy of ritual plant uses, and articulate *Azerin*-like case studies with urban ethnobiology and ethnobioconservation frameworks [46,47,48]. Such developments would help to situate the plants of *Azerin* within larger debates on biocultural diversity, social justice and the conservation of sacred species in rapidly changing urban landscapes.

## 3. Materials and Methods

### 3.1. Study Design and Research Characterization

This study adopted a qualitative, ethnodirected ethnobotanical design, focusing on the ritual use and symbolic significance of botanical species employed in *Azerin*, the funerary rite of *Candomblé Nação* Angola. An ethnodirected approach was used in the sense that the analytical categories were guided primarily by emic classifications and meanings attributed by the religious community.

The case study presented here is part of a broader ethnobotanical research program on sacred plants in *Candomblé Nação* Angola conducted in temples located in the metropolitan region of Rio de Janeiro, Brazil, during which thirty ritual events were documented through participant observation. Within this larger program, the present article reports a single, in-depth case study of the *Azerin* funeral rite observed on 10 June 2024 at the temple Abassá de Kavungo Tumba Junsara Filho. The emphasis is on the descriptive and interpretative dimensions of plant use rather than on statistical generalization. Participant observation was the central methodological strategy to document how plants are mobilized, handled, and signified during the funeral ritual.

Ethical approval for the broader research project was granted by the Research Ethics Committee of Hospital Universitário Pedro Ernesto, Universidade do Estado do Rio de Janeiro (Plataforma Brasil CAAE 81719924.0.0000.5259; opinion number 6,996,993, 20 June 2024), in accordance with Brazilian regulations for research involving human beings in the humanities and social sciences (Resolutions CNS 466/2012 and 510/2016) and national legislation on access to traditional knowledge associated with biodiversity (Law 13.123/2015). Written informed consent (Registro de Consentimento Livre e Esclarecido, RCLE) was obtained from religious leaders and interviewees before data collection, and specific authorization was negotiated with the leadership of Abassá de Kavungo Tumba Junsara Filho for participant observation and photographic documentation of the *Azerin* rite. All procedures reported in this article were conducted in accordance with the approved ethical protocol.

### 3.2. Theoretical Framework

The theoretical framework underpinning this study draws on specialized literature in Afro-Brazilian ethnobotany, the anthropology of death, and ethnoreligious studies. It includes books, theses, and peer-reviewed articles consulted through academic databases such as Google Scholar and SciELO. The main search descriptors used were “*Azerin*”, “*Mukundu*”, “*Ntambi*”, “*Axexê*”, “*Nkundu*”, “Funerary rite in *Candomblé Nação* Angola”, and “Afro-descendant ethnobotany” in Portuguese, English, and Spanish. This body of literature informed the interpretation of the ritual, the notion of sacred plants, and the categories related to spiritual protection, purification, and communication with ancestors.

### 3.3. Study Site

Fieldwork for the present case study was conducted at *Terreiro* Abassá de Kavungo Tumba Junsara Filho, a *Candomblé Nação* Angola temple located in the municipality of São João de Meriti (Baixada Fluminense; Metropolitan Region of Rio de Janeiro), state of Rio de Janeiro, Southeast Brazil. São João de Meriti is a densely urbanized lowland municipality situated within the Atlantic Forest domain, characterized by a highly anthropogenic landscape matrix. For geographic reference and comparability across studies, the settlement is located at approximately 22°48′14″ S, 43°22′20″ W (WGS84; =−22.803889, −43.372222), at 19 m a.s.l. According to the most recent official population estimate for the municipality (reference date 1 July 2024), São João de Meriti has 466,536 inhabitants.

Within the religious genealogy of *Candomblé Nação* Angola, *Abassá de Kavungo Tumba Junsara Filho* is the first lineage descending from *Terreiro Nzo de Luango Tumba Junsara Filho*, founded on 2 December 1962 by Claudionor da Silva Félix, and is currently led by Makota Omeluá. In emic terms, the local community self-identifies as povo de santo (povo de *terreiro*) of the Angola Nation (Bantu-Angola lineage). The declared religion is *Candomblé Nação* Angola. The main language used in everyday interaction and for the coordination of ritual activities is Brazilian Portuguese (Glottolog: braz1246; Portuguese macro-language: port1283); however, the liturgical register mobilizes a specialized ritual lexicon predominantly associated with Bantu-Angola traditions, as locally enacted within *Candomblé Nação* Angola.

The specific ritual observed for this article was the *Azerin* funerary rite dedicated to Mameto Getojamum, the deceased religious leader (Mameto de *Nkisi*) of this *terreiro*, performed in June 2024. The unit of analysis is the ritual as a collective performance, including its spatial organization, sequence of liturgical acts, and the plants mobilized at each stage.

### 3.4. Participant Observation and Data Collection

Participant observation was implemented as a systematic method aimed at achieving an in-depth understanding of the ritual context. For the *Azerin* case study, participant observation covered the entire temporal span of the ceremony, from the preparatory activities carried out before the arrival of the participants to the closure of the in-person ritual actions on the same day. The first author participated in the ritual as an observer integrated into the religious community, following a protocol of respectful presence agreed in advance with the leadership of the *terreiro* and in line with classic approaches to participant observation in ethnoreligious settings [60].

The *Azerin* funeral rite was attended by approximately 25 participants (n = 25), including the temple’s religious leader, senior initiates (such *as makotas, ogãs,* and *ekedis*), family members of the deceased, and other lay devotees. For the purposes of this study, particular attention was given to the actions and explanations of religious authorities and senior initiates, who are locally recognized as specialists in the management of sacred plants and ritual knowledge. In order to minimize intrusiveness in a funerary rite that is highly regulated, individual-level sociodemographic variables were not systematically elicited during the ceremony. When relevant and ethically appropriate, such variables may be reported only in aggregated form and based exclusively on self-declaration, as recorded in consent forms and/or voluntarily disclosed during prior visits. In the present case study, the available aggregated descriptors include participants’ self-identified gender distribution (women: n = 15; men: n = 10) and an age profile spanning adulthood (18–70 years).

Field notes were taken throughout the different phases of the ritual, with particular attention to: (i) the temporal sequence of actions; (ii) the spatial disposition of plants on altars, on the body of the deceased, and in other ritual structures; (iii) the modes of preparation and handling of plant material (e.g., arrangements, bundles, mats); and (iv) the emic explanations and terms used by religious authorities when referring to specific species and their ritual functions. In addition to the written field diary, the researcher recorded informal explanations and comments offered spontaneously by the leadership and senior initiates during the ritual and in previous visits to the *terreiro*. No formal semi-structured interviews were conducted specifically about the *Azerin* rite; all ethnographic information analyzed in this article derives from participant observation, field notes, and such informal emic explanations [61].

The criteria for observing and selecting plants were their ritual centrality and symbolic relevance in *Azerin*. Only species that were effectively present and used with explicit ritual intent during the funeral rite were included in the analysis; occasional plants present in the yard or temple grounds but not incorporated into the ritual liturgy were not considered. This criterion was adopted to ensure analytical focus and reproducibility [61].

In line with ethnoreligious studies, the use of participant observation allowed the researcher to access internal categories, expressions, and worldviews and to interpret plant use within the broader cosmology of *Candomblé Nação* Angola rather than as a set of isolated botanical items.

### 3.5. Botanical Documentation and Taxonomic Procedures

Given the funerary and highly regulated nature of the *Azerin* rite, botanical documentation followed a minimally invasive, two-step protocol. First, all species visibly employed in the ceremony were recorded in situ through high-resolution photographic images taken immediately before the beginning of the ritual and, when authorized by the religious leadership, during specific liturgical moments. No destructive sampling or manipulation of plant material was carried out inside the sacred space in order to avoid interference with the rite and to comply with religious prescriptions concerning sacred plants. Vernacular names, salient morphological traits, and ritual attributions were registered in field notebooks in dialog with the priesthood and senior initiates [62].

In a second step, for each taxon recognized in the ritual context, reference specimens were collected outside the core ritual area, in the vegetated sectors (“mato space”) associated with the participating *terreiros*, following standard botanical procedures for the collection, pressing, and drying of fertile material [63,64]. The resulting voucher specimens were deposited in the Herbarium of the Rio de Janeiro Botanical Garden (RB), where they are available for future consultation and taxonomic revision. Photographic records were used in parallel to document diagnostic characters and to register the spatial arrangement and forms of use of each species in the ritual setting.

Botanical identification was performed using specialized floristic and taxonomic literature, complemented by consultation of online herbarium databases, particularly JABOT (Reflora) [65] and SpeciesLink [66]. Scientific names and family circumscriptions followed the Angiosperm Phylogeny Group IV [67]. The spelling and current taxonomic status of native and exotic species were verified in the Flora e Funga do Brasil database [68] and in The World Flora Online [69]. For each species, the following information was systematized in a data matrix and subsequently summarized in a table: family, scientific name, vernacular name, and main ritual function in *Azerin*.

### 3.6. Qualitative Data Analysis

Data analysis proceeded in two complementary stages. First, all field notes and photographic records were reviewed to reconstruct the sequence of the *Azerin* rite and to map the spatial and functional distribution of the plants throughout the ritual. In this stage, a descriptive matrix was created, associating each plant species with (i) its physical location in the ritual space; (ii) the liturgical act in which it was employed (e.g., preparation of the ancestral altar, protection of the body, delimitation of sacred space); and (iii) the emic categories of meaning attributed to it (e.g., protection, purification, connection with the ancestors, representation of the deceased).

Second, a thematic content analysis was applied to the ethnographic material, combining inductive coding, derived from the statements and practices observed in the field, with deductive coding, based on the theoretical literature on sacred plants and Afro-Brazilian funerary rites. The main analytical axes were (a) ritual functions of each plant in relation to the passage from *Àiyé* to *Òrun*; (b) forms of spiritual protection and containment of *Eguns* associated with plant structures; and (c) ways in which plant use materializes cosmological concepts of life, death, and return to nature in *Candomblé Nação* Angola.

The final synthesis sought to integrate botanical, symbolic, and ritual dimensions, providing an interpretative framework that connects the observed species to the broader universe of Afro-Brazilian traditional knowledge and to ongoing discussions in ethnobotany and ethnobiology.

## 4. Conclusions

The ritual ceremonies that permeate the universe of *Candomblé* hold undeniable importance, but it is *Azerin* that stands out for its uniqueness and spiritual relevance. This rite is particularly significant, as its purpose is to release the *Egun* from inert matter, a task that carries considerable weight for the priests who undertake it. Understanding this practice is essential, as it involves not only saying farewell to a loved one but also celebrating the continuity of life in an endless cycle that interweaves the worlds of the living and the dead.

Thus, the death of an initiate demands the performance of funeral rites, which are carried out with reverence and respect. The funeral rite of the Angola Nation, known as *Azerin*, holds a prominent position in the ritualistic landscape of *Candomblé*, presenting itself as a moment of profound spiritual reflection. In this rite, the presence of deities such as Zumbarandá, Kavungo, and Matamba is indispensable. Although Zumbarandá and Kavungo maintain a close connection with the *Azerin*, it is Matamba, queen and matriarch of the *Eguns*, who presides over the ceremony.

During the execution of the *Azerin*, the *Eguns*, or ancestral spirits, occupy a central role, especially those who held important social or religious roles, such as priests. The inherent hierarchy of *Candomblé* is revealed not only in the conduct of the rituals but also in the social structure they imply. *Azerin* is not limited to death; it also encompasses life and the dynamic relationship between the living and their ancestors, reinforcing the connection that sustains the cultural and spiritual identity of the community.

In this context, ritual plants are selected for their associations with deities and their spiritual protective properties, with each plant species holding specific meanings. This intimate connection with nature highlights the importance of natural elements in strengthening *axé*, the vital energy that permeates the entire rite.

Consequently, throughout the ritual, the use of five sacred plant species becomes evident, varying between protection against *Eguns* and the physical representation of the deceased. These plants not only adorn the rite but also become essential instruments in the conduction of *axé*, being used in herbal preparations (*Amassi*), fumigations, and in the protection of participants.

Thus, the funeral rite is revealed as a sacred space where ancestral memory materializes, establishing *Azerin* as an affirmation of the continuity of life. Through this rite, the life-death-life cycle is perpetuated, reaffirming the importance of returning to nature and the ongoing experience of spirituality that connects the living to their ancestors.

## Figures and Tables

**Table 1:** List of species used during the *Azerin* rite.

Family (Genus/Species)	Scientific Name (Herbarium Voucher)	Local Name	Ritual Use
ARECACEAE (2/2)	*Elaeis guineensis* Jacq (RB896048)	Dendezeiro	Leaves of the African oil palm is used in the preparation of the ancestral altar. They is also used to prevent *Eguns* from approaching.
	*Raphia vinifera* P.Beauv. (RB896052)	Palha-da-costa	Used to wrap around the left wrist of the *Ogãs* present during the rite and the heads of the women in attendance to prevent *Eguns* from approaching.
ASPARAGACEAE (1/1)	*Dracaena fragrans* (L.) Ker Gawl. (RB896049)	Dracena; Peregum	Used for spiritual cleansing at the end of the ritual.
CUCURBITACEAE (1/1)	*Lagenaria siceraria* (Molina) Standl. (RB896051)	Cabaça	The dried calabash fruit is cut in half and placed in a clay bowl to be played using a mulberry branch, guiding the ritual and replacing the Atabaque. It represents the physical body of the deceased.
MORACEAE (1/1)	*Morus nigra* L. (RB896050)	Amora	The mulberry branch is used to strike the calabash.

## Data Availability

The data supporting the findings of this study are available from the corresponding author upon reasonable request. The raw data are not publicly available in order to safeguard participants’ rights and privacy, in accordance with ethical guidelines and applicable legislation.
